# Compliance with 24-h Movement Behaviour Guidelines among Belgian Pre-School Children: The ToyBox-Study

**DOI:** 10.3390/ijerph15102171

**Published:** 2018-10-03

**Authors:** Marieke De Craemer, Duncan McGregor, Odysseas Androutsos, Yannis Manios, Greet Cardon

**Affiliations:** 1 Department of Movement and Sports Sciences, Ghent University, 9000 Ghent, Belgium; Greet.Cardon@UGent.be; 2 Department of Health and Community Sciences, Glasgow Caledonian University, Glasgow G1 2FF, UK; Duncan.McGregor@gcu.ac.uk; 3 Department of Nutrition and Dietetics, School of Health Sciences & Education, Harokopio University, 17676 Athens, Greece; oandrou@hua.gr (O.A.); manios@hua.gr (Y.M.)

**Keywords:** pre-school children, physical activity, sedentary behaviour, sleep, movement behaviours, 24-h guidelines, compliance

## Abstract

The 24-h day—containing physical activity, sedentary behaviour and sleep—in pre-school children has not yet been extensively investigated. The aim of the current study was to investigate pre-schoolers’ compliance with the 24-h movement behaviour guidelines (i.e., three hours/day total physical activity, a maximum of one hour/day of screen time and 10–13 h sleep/night). In total, 595 pre-schoolers (53.3% boys, mean age: 4.2 years) provided complete data for the three behaviours. Physical activity was objectively measured with accelerometers, while screen time and sleep were parent-reported through questionnaires. The proportion of pre-schoolers complying with the 24-h movement behaviour guidelines was calculated on weekdays and on weekend days. Low compliance rates were found: 10.1% on weekdays and only 4.3% on weekend days. The majority of pre-schoolers complied with the sleep duration guidelines (>90% on weekdays and weekend days), followed by the screen time guidelines (61% on weekdays and 28% on weekend days). The lowest compliance rates were found for physical activity (<20% on weekdays and weekend days). Overall, low percentages of pre-schoolers complying with the 24-h movement behaviour guidelines were found, and the lowest compliance was found for physical activity.

## 1. Introduction

Engaging in sufficient amounts of physical activity, trying to limit sedentary time and having a sufficient amount of sleep are important for health [1]. Previous research has separately investigated physical activity, sedentary behaviour, and sleep in pre-school children. This was reflected in the establishment of physical activity guidelines (i.e., 180 min of total physical activity per day), screen time guidelines (i.e., no more than one hour of screen time per day), and sleep guidelines (i.e., sleep for 10–13 h per night, including nap times) for pre-school children [2,3,4,5,6]. More recent evidence showed that these movement behaviours (i.e., physical activity, sedentary behaviour and sleep) interact with each other [7,8,9]. Therefore, emphasis has been put on an integrated approach to healthy movement behaviours in pre-school children, which resulted in the establishment of new evidence-informed 24-h movement behaviour guidelines in this age group in both Canada and Australia [10,11]. Following the recommendations, a healthy 24-h day should contain (1) at least 180 min of physical activity, of which 60 min are spent in energetic play; (2) a maximum of one hour of sedentary screen time and not being restrained/sedentary for more than one hour at a time; and (3) 10–13 h of good quality sleep [10].

It is important to investigate the proportion of pre-school children complying with these newly established guidelines to inform researchers which percentage of pre-schoolers already engage in healthy 24-h days. To our knowledge, only two recent studies in Canada and Australia have explored the compliance with the 24-h movement behaviour guidelines in pre-school-aged children. In Canada, only 12.7% of 803 pre-school children (mean age: 3.5 years) adhered to the 24-h movement behaviour recommendations, with a high proportion meeting the sleep duration and physical activity guidelines, and only a limited percentage meeting the screen time recommendation [1]. Similar results were found in 248 Australian pre-school children (mean age: 4.2 years), with only 14.9% of pre-schoolers meeting all three guidelines. Again, a higher proportion of pre-school children met the physical activity and sleep duration guidelines, whereas only few complied with the screen time recommendation [12].

To the best of our knowledge, no study has been conducted in Europe investigating the proportion of pre-school children complying with the newly established guidelines. Therefore, the aim of the current paper was to describe Belgian pre-schoolers’ compliance to the individual (i.e., each movement behaviour separately) and integrated (i.e., all three behaviours together) 24-h movement behaviour guidelines for the early years. As behaviours might be different between weekdays and weekend days [13,14], compliance with the movement behaviour guidelines will be explored for weekdays and weekend days.

## 2. Materials and Methods

### 2.1. Study Protocol

Participants in the present study were part of the baseline measurements within the European ToyBox-study (www.toybox-study.eu). A detailed description of the ToyBox-study was published elsewhere [15,16]. In brief, the ToyBox-study aimed at preventing pre-school children from becoming overweight or obese by developing and testing a kindergarten-based, family-involved intervention in six European countries (Belgium, Bulgaria, Germany, Greece, Poland, and Spain). This study only used the Belgian baseline data, as only the Belgian pre-school children wore accelerometers to measure their physical activity levels, while pedometers were used in the other European countries. This study was approved by the Ethical Committee of Ghent University Hospital (EC/2010/037).

All municipalities were listed within the provinces of Western and Eastern Flanders (Belgium), and information on their socio-economic status (SES) was provided (years of education for the population of 25–55 years (cut-off: >14 years of education) or annual income (quantitative variable)). Based on the selected SES variables, tertiles were created and five municipalities per SES status were randomly selected by the coordinating centre (i.e., Harokopio University Athens, Kallithea, Greece). Subsequently, pre-schools within these municipalities were randomly selected (with the exclusion of the 20% of pre-schools with the smallest number of pupils). Eventually, 27 Flemish pre-schools participated in this study. All parents of pre-schoolers that were born between 2007 and 2008 received an information letter about the study and only children whose parents provided informed consent participated in the study. Data collection occurred between May and June 2012 and consisted of pre-schoolers wearing an accelerometer and their parents filling in a questionnaire (Principal Caregiver’s Questionnaire).

### 2.2. Measurements

#### 2.2.1. Physical Activity

Pre-schoolers’ physical activity was measured using three ActiGraph (ActiGraph corporation, Pensacola, FL, USA) accelerometer models, namely the GT1M, the GT3X, and the GT3X+. It is acceptable to use these activity monitors in one study, as there is a strong agreement regarding vertical axis counts between these three types of ActiGraph accelerometers [17]. In addition, the GT1M accelerometer has been validated to measure physical activity in pre-school children [18]. Furthermore, only the vertical axis output was used in the present study. Using ActiLife 5.5.5-software (ActiGraph corporation, Pensacola, FL, USA), accelerometers were initialised to measure activity counts in 15 s epochs, taking into account pre-schoolers’ intermittent patterns of movement [19]. Accelerometers were worn on the right hip, secured by an elastic waist band. The participating pre-school children were asked to wear the accelerometer for six consecutive days (including two weekend days) during all waking hours, which means that they were asked to wear the accelerometer from waking up until going to bed. They had to remove the device for water-based activities and for sleeping. Pre-schoolers’ parents were given an information letter with instructions on how to handle the device, to ensure that the device was worn correctly. After data collection, accelerometers were downloaded using ActifLife 5.5.5-software (ActiGraph Corporation, Pensacola, FL, USA) and data files were then reduced using Meterplus version 4.3 software (Santech Inc., San Diego, CA, USA). Data from both the first (i.e., fitting day—done by the researchers) and sixth day (i.e., collection day) were omitted, because the data of these days were incomplete. Periods of ten minutes or more of consecutive zeros were deleted, as these periods were regarded as non-wearing time. To be included in the analyses, pre-school children were required to have at least six hours of accelerometer recordings on a minimum of three days, including one weekend day [20]. Minutes of total physical activity on weekdays and weekend days were afterwards categorised using the cut-point of 275 counts/15 s of Reilly et al. [21]. Overall physical activity was calculated as follows: ((physical activity on weekdays) × 5) + (physical activity on weekend days) × 2)/7. To calculate the proportion of pre-school children complying with the physical activity guideline of being physically active for more than 180 min per day, minutes of total physical activity were dichotomised into 0 (<180 min of total physical activity per day) and 1 (≥180 min of total physical activity per day).

#### 2.2.2. Screen Time

Television viewing and computer use were assessed separately by two questions in the Principal Caregiver’s Questionnaire, which has been shown to be a reliable questionnaire [22]. Each of these was assessed separately in respect of weekdays and weekend days. For television viewing, the question was formulated separately for weekdays and weekend days as follows: “About how many hours a day does your child usually watch television (including DVDs and videos) in his/her free time?”. Answer possibilities were “never”, “less than 30 min/day”, “30 min to <1 h/day”, “1–2 h/day”, “3–4 h/day”, “5–6 h/day”, “7–8 h/day”, “8 h per day”, “more than 8 h/day”, and “I don’t know”. For computer use, the question was also formulated separately for weekdays and weekend days as follows: “About how many hours a day does your child use the computer for activities like playing games on a computer, game consoles (e.g., PlayStation, Xbox, GameCube) during leisure time?”. Answer possibilities were identical to the television viewing questions. Answer possibilities were recoded into minutes of television viewing and computer playing per day by using the midpoint method [13], and were then added up to reflect the total screen time. Overall screen time was calculated as follows: ((screen time on weekdays) × 5) + (screen time on weekend days) × 2)/7. To calculate the proportion of pre-school children complying with the screen time recommendation of less than one hour of screen time per day, minutes of total screen time were dichotomised into 0 (>60 min of screen time per day) and 1 (≤60 min of screen time per day).

#### 2.2.3. Sleep Duration

Sleep duration on weekdays and weekend days were each assessed by one question in the Principal Caregiver’s Questionnaire, formulated separately for weekdays and weekend days as follows: “How many hours of sleep does your child usually have during the night?”. Answer possibilities were “less than 6 h”, “6–7 h”, “8–9 h”, “10–11 h”, “12–13 h”, “14 h”, “more than 14 h”, and “I don’t know”. Answer possibilities stating “10–11 h” and “12–13 h” were recoded into 1, reflecting all pre-school children complying with the sleep duration guidelines of 10–13 h of sleep per night. Answer possibilities stating sleep duration shorter or longer than 10–13 h of sleep per night were recoded into 0, reflecting all pre-school children not complying with the sleep duration recommendations. To calculate overall sleep duration, answer possibilities were recoded into minutes of sleep by using the midpoint method [13]. Afterwards, overall sleep duration was calculated as follows: ((sleep duration on weekdays) × 5) + (sleep duration on weekend days) × 2)/7.

### 2.3. Statistical Analysis

Descriptive characteristics are presented as means and standard errors for continuous variables and as percentages for categorical variables. Descriptive statistics were used to examine the average time spent on total physical activity, screen time and sleep, for weekdays, weekend days, and overall, respectively. Furthermore, the proportion of pre-school children complying with the 24-h movement behaviour guidelines was calculated, and also for each movement behaviour separately and all other possible combinations. Multilevel regression analyses with three levels (pre-school child; class; kindergarten) were performed to assess differences between boys and girls regarding age, body mass index, total physical activity, screen time and sleep duration. In addition, to compare pre-school children with valid data to pre-school children who did not have valid data, attrition analyses were conducted as a logistic regression analysis with three levels (pre-school child; class; kindergarten). Statistical significance was set at a *p*-value of <0.05.

## 3. Results

In total, 1082 pre-schoolers wore an accelerometer during the measurements. Out of those 1082 pre-school children, 867 (80.1%) had valid data for a minimum of three days including one weekend day. Only 768 (72.6%) of those pre-schoolers had data on sleep and screen time, and 595 pre-schoolers (55.0%) were between three and four years old. Therefore, the final sample included 595 Belgian pre-school children (53.3% boys, mean age: 4.20 ± 0.46; 215 3-year-olds, 371 4-year-olds) who had valid data for all outcome variables (i.e., physical activity, sedentary behaviour and sleep) and were therefore included in the analyses. In Table 1, descriptive statistics of the participants can be found. Pre-school children had an average monitor wear time per day of 10.75 (±1.37) hours/day and had on average 3.79 (±0.41) valid days. In the total sample, children were physically active for an average of 134.17 (±36.24) minutes per day, spent approximately 80.80 (±54.12) minutes on television viewing or computer playing, and had an average sleep duration of 10.99 (±1.02) hours per night. No significant differences were found between boys and girls regarding these three movement behaviours. A difference was found for the average amount of steps per day, with boys taking more steps compared to girls on weekdays (*p* < 0.001) and weekend days (borderline significant, *p* = 0.08). Attrition analyses showed that younger pre-school children were more likely to have incomplete data compared to older pre-school children (Odds Ratio (OR) = 1.88; 95% CI = 1.39–2.54); no differences were found between pre-school boys and pre-school girls (OR = 99; 95% CI = 0.77–1.30).

**Figure 1:**
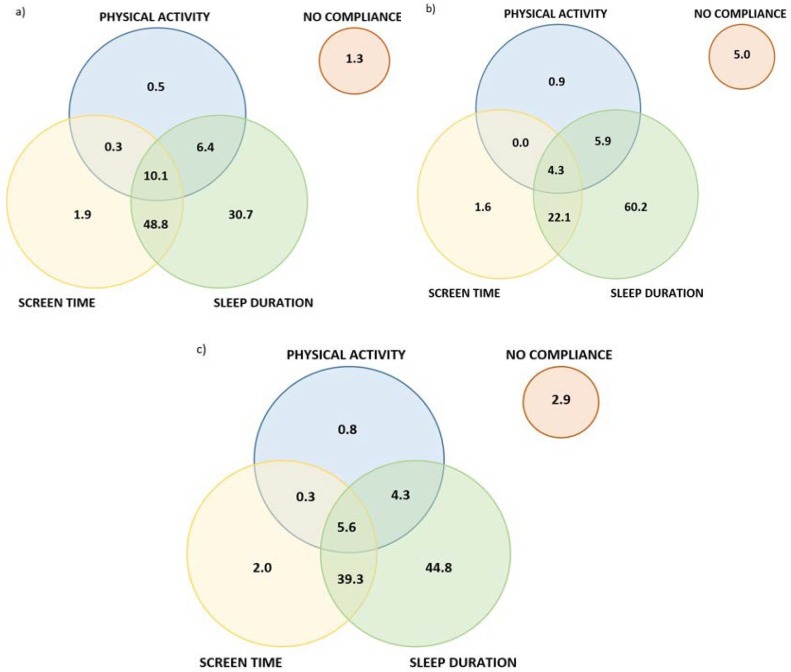
Venn diagrams showing the percentage of pre-school children (not) complying with the recommendations for physical activity, screen time, and sleep duration, and combinations of these recommendations, for (**a**) weekdays; (**b**) weekend days; and (**c**) overall. The sum of each circle is equivalent to the percentage of pre-school children meeting the individual recommendations (e.g., 17.3% for physical activity on weekdays).

Figure 1 displays the percentage of pre-school children complying with the 24-h movement behaviour guidelines on weekdays, weekend days, and overall, including the proportion of pre-school children complying with the separate recommendations for physical activity, screen time and sleep duration, and combinations of these recommendations. A total of 10.1% of Belgian pre-school children complied with the 24-h movement behaviour guidelines on weekdays, while this proportion was much lower on weekend days (4.3%) and overall (5.6%). Only 1.5% (*n* = 9) of all participants complied with the 24-h guidelines both on weekdays and weekend days. The majority of pre-schoolers complied with the sleep duration guidelines (96.0% on weekdays, 92.5% on weekend days, 94.0% overall), followed by the screen time guidelines (61.1% on weekdays, 28.4% on weekend days, and 47.2% overall). Few pre-schoolers complied with the physical activity guidelines (17.3% on weekdays, 10.8% on weekend days, and 11.0% overall). Overall, 1.3%, 5.0%, and 2.9% of the children did not comply with any of the three recommendations on weekdays, weekend days, and overall, respectively.

## 4. Discussion

The aim of the current study was to investigate the proportion of Belgian pre-school children complying with the individual (i.e., for physical activity, screen time, and sleep duration) and integrated 24-h movement behaviour guidelines for the early years. Compliance was described for weekdays and weekend days separately, and overall.

Overall, pre-schoolers’ compliance with the integrated 24-h movement behaviour guidelines was low. Only 10.1% of pre-schoolers complied with the 24-h movement behaviour guidelines on weekdays, and an even lower percentage (4.3%) was found on weekend days. To our knowledge, only two other studies researched pre-schoolers’ adherence with the individual and integrated 24-h movement behaviour guidelines for the early years [1,12]. The Canadian study conducted a study with 803 three- to four-year-old pre-school children, of which 12.7% complied with the 24-h movement behaviour guidelines. The highest compliance was found for sleep duration, and the lowest compliance was found for screen time [1]. The Australian study (248 four-year-old pre-school children) found similar results, with 14.9% of 248 pre-school children complying with the 24-h recommendations. In the latter study, the highest adherence was found for physical activity, and the lowest adherence was found for screen time [12]. Up to some point, results from the current study are comparable with the Australian and Canadian studies. A very large proportion of pre-schoolers adhering to the sleep duration guidelines were found (96.1% on weekdays, 92.5% on weekend days), which is comparable with the Australian (88.7%) and Canadian studies (83.9%). However, compliance with physical activity recommendations was rather low (17.6% on weekdays, 11.1% on weekend days), which was a major reason for the low proportion of children complying with the 24-h recommendations. Strikingly, much higher compliance with physical activity recommendations were found in the Australian (93.1%) and Canadian study (61.8%). These higher compliance rates with the physical activity recommendations were also found in European studies looking at pre-schoolers’ compliance with 180 min of physical activity per day [23,24]. For example, all United Kingdom pre-school children complied with the physical activity guidelines in the study of Hesketh et al. (2014), and 95.4% of all activity observations in the study of Collings et al. (2017) were higher than 180 min of physical activity per day. These differences in compliance rates with the current study might be explained by the use of different measurement instruments and accordingly different cut-points. For example, Actigraph accelerometers were used in the Australian study, but a combination of two cut-points (i.e., Evenson et al. and Pate et al.) was used to calculate the compliance with the 24-h movement behaviour guidelines [12,25,26]. Furthermore, Actical accelerometers were used in the Canadian study and another set of cut-points [27] was used to process the data [1]. In the current study, the accelerometer cut-point from Reilly et al. (2003) was used to make a distinction between sedentary behaviour and physical activity [21]. This cut-point is higher than those found in other accelerometer calibration studies in pre-school children [21,25,26] and with those used in the Australian and Canadian studies [1,12]. However, the cut-points of Pate et al. (2006) and Evenson et al. (2008), used in these other studies relied on structured activities to calibrate their accelerometers [25,26], but structured activities do not reflect pre-schoolers’ intermittent patterns of physical activity behaviour [19]. In contrast, the calibration of the cut-point of Reilly et al. (2003) was based on direct observation of the free-living activities of pre-school children, which is a common criterion measure in assessing pre-schoolers’ physical activity [28] and should better reflect their patterns of physical activity behaviour. For that reason, we decided to use the cut-point of Reilly et al. (2003) [21].

The current study is the first study conducted within Europe in which pre-schoolers’ compliance with the 24-h movement behaviour guidelines was investigated. Another added value of the current study is the insight into pre-schoolers’ compliance on weekdays and weekend days. Data showed that compliance is higher on weekdays compared to weekend days. The proportion of pre-schoolers not complying with any of the guidelines is larger on weekend days compared to weekdays. These data suggest that more effort should be done to increase compliance on weekend days. During weekend days, pre-school children are at home, which calls for a home-based or family-based approach in increasing the adherence on weekend days. However, most interventions are currently school-based, and as compliance on weekdays is also low, it is still important to target weekdays as well, which might be done through schools. Furthermore, other studies already showed that pre-school girls engage in lower levels of physical activity and are less physically active in general compared to pre-school boys [29,30,31]. This fact should be considered in developing future interventions aiming at improving compliance with the 24-h movement behaviour guidelines. For example, interventions should provide both activities aimed at pre-school girls (e.g., activities at light physical activity levels) and activities aimed at pre-school boys (e.g., activities at moderate or high physical activity levels).

As the idea of the 24-h continuum in pre-school children recently received more attention, especially since the launch of the 24-h movement guidelines for the early years [10], scientific literature regarding a healthy 24-h day in pre-school children is currently limited. Future studies should explore data and adherence to the 24-h guidelines and associations with individual and environmental determinants, and with health consequences to get a better insight and to inform future interventions targeting the 24-h continuum. In addition, objective measurements of those 24-h days in pre-school children, containing objective measurements of all three behaviours (i.e., sleep, sedentary behaviour, and physical activity), might give us a good insight into the composition of those three behaviours in this age group. Currently, the 24-h movement behaviour guidelines do not yet include a proportion of sedentary behaviour, but only use guidelines regarding screen time. However, the use of objective measurement devices across 24 h might help to set guidelines regarding a maximum proportion of sedentary behaviour per day as well. Using ActivPAL measurement devices to monitor and measure this 24-h day might be one of the best options to do this, as it is currently one of the only non-obtrusive measurement devices that can objectively measure all three behaviours during several consecutive days. In addition, it has already been validated in pre-school children [32,33,34], and it would eliminate the accelerometer cut-point problem in pre-school children. Future studies should try to use the same measurement devices and accordingly the same steps in data processing to increase comparability across studies.

A strength of the current study includes the large amount of data on Belgian pre-schoolers’ 24-h movement behaviours. Furthermore, physical activity was objectively assessed with the use of accelerometers. A limitation might be the subjective parental report of screen time and sleep duration, which might lead to a possible bias because of parents’ social desirability. Additionally, accelerometer data from pre-school children were included if they had a minimum of six hours of valid wear time per day, which means that activities during some parts of the day might not have been captured. Finally, we acknowledge that the sample used in the current study is not completely generalizable for all Belgian children, because pre-school children were only sampled in specific regions in Flanders, the Dutch-speaking part of Belgium (i.e., Western and Eastern Flanders). However, the procedure used in the current study tried to give a fair approximation of the average situation by including pre-schoolers of both low, medium and high SES backgrounds and in each kindergarten (almost) complete classes were included.

## 5. Conclusions

Overall, low compliance with the 24-h movement behaviour guidelines was found in Belgian pre-school children. Physical activity was the behaviour that was least complied with, and thus most attention should be paid to increase pre-schoolers’ physical activity. It is possible that previous studies investigating pre-schoolers’ compliance with the 24-h movement behaviour guidelines overestimated compliance, especially regarding pre-schoolers’ physical activity. However, it must be acknowledged that over- and underestimation of compliance with physical activity guidelines is possibly directly related to the choice of accelerometer cut-points.

## Figures and Tables

**Table 1:** Descriptive characteristics of the sample.

	Total Sample	Boys	Girls	*p*-Value
	(*n* = 595)	(*n* = 317)	(*n* = 278)	
Age (years)	4.20 (0.02)	4.21 (0.02)	4.20 (0.02)	0.25
BMI (kg/m^2^)	15.88 (0.05)	15.95 (0.07)	15.79 (0.08)	0.14
Weight status (%)				0.9
Underweight	10.5	10.7	10.2	
Normal weight	79	79.6	78.4	
Overweight/obese	10.5	9.7	11.3	
Total physical activity (min/day)	133.74 (1.49)	135.81 (2.04)	131.36 (2.17)	0.13
Weekday	145.50 (1.62)	147.08 (2.21)	143.71 (2.36)	0.3
Weekend day	121.94 (1.94)	124.56 (2.64)	119.00 (2.82)	0.15
Steps per day				
Weekday	10,543.31 (156.49)	10,983.57 (172.76)	8210.98 (217.23)	<0.001
Weekend day	8022.41 (187.36)	10,053.66 (177.01)	7809.25 (224.09)	0.08
Screen time (min/day)	80.31 (2.24)	80.31 (3.07)	80.32 (3.25)	0.99
Weekday	65.15 (2.07)	64.63 (2.81)	65.72 (2.99)	0.79
Weekend day	119.50 (3.42)	121.71 (4.68)	117.03 (4.95)	0.49
Sleep duration (h/day)	10.99 (0.04)	10.99 (0.06)	11.01 (0.06)	0.85
Weekday	10.95 (0.04)	10.95 (0.06)	10.95 (0.06)	0.99
Weekend day	11.12 (0.05)	11.11 (0.07)	11.14 (0.07)	0.7
